# Evaluation of the Quality of Selected White and Red Wines Produced from Moravia Region of Czech Republic Using Physicochemical Analysis, FTIR Infrared Spectroscopy and Chemometric Techniques

**DOI:** 10.3390/molecules28176326

**Published:** 2023-08-29

**Authors:** Iwona Budziak-Wieczorek, Vladimír Mašán, Klaudia Rząd, Bożena Gładyszewska, Dariusz Karcz, Patrik Burg, Alice Čížková, Mariusz Gagoś, Arkadiusz Matwijczuk

**Affiliations:** 1 Department of Chemistry, Faculty of Life Sciences and Biotechnology, University of Life Sciences in Lublin, Akademicka 15, 20-950 Lublin, Poland; 2 Department of Horticultural Machinery, Mendel University in Brno, Valtická 337, 691 44 Lednice, Czech Republic; vladimir.masan@mendelu.cz (V.M.); patrik.burg@mendelu.cz (P.B.); alice.cizkova@mendelu.cz (A.Č.); 3 Department of Biophysics, University of Life Sciences in Lublin, Akademicka 13, 20-950 Lublin, Poland; klaudia.rzad@up.lublin.pl (K.R.); bozena.gladyszewska@up.lublin.pl (B.G.); 4 Department of Chemical Technology and Environmental Analytics, Krakow University of Technology, 31-155 Krakow, Poland; dariusz.karcz@pk.edu.pl; 5 ECOTECH-COMPLEX—Analytical and Programme Centre for Advanced Environmentally-Friendly Technologies, Maria Curie-Sklodowska University, Głęboka 39, 20-033 Lublin, Poland; 6 Department of Biochemistry and Molecular Biology, Medical University of Lublin, 20-093 Lublin, Poland; mariusz.gagos@umlub.pl

**Keywords:** FTIR spectroscopy, chemometric analysis, principal component analysis, grape varieties, wine samples

## Abstract

The FTIR-ATR method coupled with the multivariate analysis of specific spectral areas of samples was developed to characterize two white grape varieties (Sauvignon Blanc and Hibernal) and two blue grape varieties (André and Cabernet Moravia) of wine planted and harvested in the Moravia region, Czech Republic. Principal component analysis and hierarchical cluster analysis were performed using fingerprint regions of FTIR spectra for all wines. The results obtained by principal component analysis in combination with linear discriminant analysis (PCA-LDA) scores yielded clear separation between the four classes of samples and showed very good discrimination between the wine samples, with a 91.7% overall classification rate for the samples. The conducted FTIR spectroscopy studies coupled with chemometrics allowed for the swift analysis of multiple wine components with minimal sample preparation. These methods can be used in research to improve specific properties of these wines, which will undoubtedly enhance the quality of the final wine samples obtained.

## 1. Introduction

The evaluation of food products’ quality often requires both the determination of the chemical composition and the characterization of physical parameters such as mechanical, rheological and thermophysical properties [1,2]. According to Johnson [3], wine is an alcoholic beverage for the production of which grapes are used. Their chemical composition ensures the course of the fermentation process, in which yeast participates. Yeast is involved in the consumption of carbohydrates in the grapes, which they convert into alcohol and carbon dioxide. Boulet et al. [4] state that wine is a complex product with valuable biological and organoleptic properties.

Jones-Moore et al. [5] state that the properties and quality of wine are the result of synergistic interactions among factors such as the variety, the quality parameters of the harvested grapes and the processing technology, yeast strains, bacteria, etc. The mentioned factors also impact the composition of wine, which consists of water (70–90%), alcohol (8–20%), acids (0.3–1%), carbohydrates (0.1–20%), pigments, phenols, minerals, vitamins, etc. [6]. Established methods for the determination of these compounds are generally based on either colorimetric or chromatographic techniques, such as gas chromatography or high-performance liquid chromatography (HPLC).

These techniques can be relatively complex, requiring sample preparation and even chemical manipulations, which makes them time-consuming, laborious and expensive. In addition, they often allow the detection of only one group of compounds. Infrared spectroscopy therefore offers a suitable alternative to conventional chemical analyses. FTIR is a non-destructive technique that provides information on the structural properties of a wide range of compounds. Among its main advantages are the response speed, a high level of automation, good resolution, environmental friendliness and overall economy of determination [7,8]. These characteristics, together with the improvements achieved by chemometrics, provide an interesting analytical tool for routine qualitative and quantitative analysis, widely used in many industries during control processes [9]. In fact, FTIR has proven to be a useful and reliable technique in the analysis of a large variety of samples in various industries, including the agri-food sector [10,11].

In oenology, FTIR analysis has been described as a routine procedure enabling the determination of classical oenological parameters [12], organic acids [13], aroma precursors [14] and phenolic compounds [15] in grapes and wines. The use of the attenuated total reflectance (ATR) accessory in FTIR studies enables the direct analysis of liquids in a simple and non-destructive manner with sufficient sensitivity [16]. Thus, ATR-FTIR has proven to be a suitable method for the analysis of liquid foods such as vinegar [17], olive oil, wine, milk or honey [9,18,19,20].

The aim of this study was to evaluate the use of FTIR as an analytical technique combined with multivariate data analysis techniques for the comparison and classification of four wine samples produced in the Moravia region in the Czech Republic. Based on the differences between the FTIR spectra, by multivariate data analysis (principal component analysis, hierarchical clustering analysis and linear discriminant analysis), we identified the specific discrimination factors useful to identify the main sources of variation between the grape varieties, as well their sweetness, in a rapid and non-destructive manner. Moreover, FTIR spectroscopic measurements were taken at three different time intervals to monitor the aging of the samples during storage.

**Legend:**
Andre wine (AW)—red grape variety
Cabernet Moravia wine (CMW)—red grape variety
Hibernal wine (HW)—white grape variety
Sauvignon blanc wine (SW)—white grape variety
**FTIR spectroscopic measurement dates:**
I—22.08.22
II—21.10.22
III—25.11.22

## 2. Results and Discussion

### 2.1. The Determination of Basic Analytical Values in Wine

The quality of varietal wines is defined mainly by their sensory attributes. These attributes are determined by physical and chemical properties. In addition to the variety itself, the quality of the processed grapes is an important factor, which is influenced by the geographical origin, ripening stage, harvest date and oenological procedures, as well as the chemical and textural parameters and the technological procedures of wine production and storage. In terms of the basic analytical parameters, overall differences can be seen between samples of white and red wines. In the case of red wines, these relate to the higher alcohol content and lower content of titratable acids (reduction linked to malolactic fermentation; at the same time, higher lactic acid content is evident), as well as a higher pH value and glycerol content.

The overview of values in Table 1 shows that the content of alcohol and glycerol corresponds to the sugar content of the must after pressing. This was in the range of 20.8–21.1 for white varieties and in the range of 21.3–22.2° NM for blue varieties. The alcohol content of the evaluated wine samples ranged between 12.19 and 13.58% and the glycerol content from 7.66 to 9.81 g·L^−1^. Michlovský [2] states that the total content of alcohol and glycerol is related to the total initial sugar content of the must, the presence of yeast during fermentation and its temperature course. From the values that he provides, it is clear that the alcohol content in white wines is in the range of 11–13% and that in red wines is 12.0–14.5%. The glycerol content in white wines is normally around 6 g·L^−1^ and in red wines usually above 10 g·L^−1^. Jones et al. [21] state that, in recent years, the content of both alcohol and glycerol in wines has shown an increasing trend, the cause of which is a number of factors, related, in particular, to climate change. The pH value is also an important indicator of wine quality. It varied between 3.13 and 3.48 for individual wine samples in this study. Michlovský [2] states that in Central European conditions, 3.10–3.30 for white wines and 3.20–3.40 for red wines can be considered a normal pH value. The content of titratable acids in the evaluated samples ranged from 4.74 to 6.58 g·L^−1^. The acid content has a complex effect on a number of wine parameters. The greatest importance in this direction is to ensure the sufficient microbiological and chemical stability of wine with an overlap in its resulting organoleptic properties [22]. Carrau et al. [23] state that the optimal content of titratable acids in white wines ranges from 5 to 7 g·L^−1^. Jackson [22] reports that the optimal concentration of total titratable acids for white wines ranges from 5.5 to 8.5 g·L^−1^ and for red wines from 4.7 to 5.1 g·L^−1^.

Acetic acid content was measured within the range of 0.04–0.40 g·L^−1^. Thus, none of the evaluated wine samples exceeded the limit value of 0.50 g·L^−1^, which is considered as the threshold at which wines can start to show negative notes—for example, the taste of sauerkraut [24]. The maximum limit for acetic acid defined by the reference method of Commission Regulation EEC 2676/90 is 1 g of acetic acid per liter of wine, which is also the sensory detection threshold [25].

The tartaric acid content of wine samples ranged from 1.83 to 2.31 g·L^−1^. The Organization Internationale de la Vigne et du Vin (OIV) states the minimum content limit of tartaric acid at the level of 0.3 g·L^−1^ for white and rosé wines and 0.4 g·L^−1^ for red wines.

The content of malic acid in the monitored wine samples ranged from 0.35 to 4.01 g·L^−1^. The malic acid content is variable, depending on the biochemical processes during fermentation and wine aging [26].

Lactic acid is mainly produced by a process called malolactic fermentation from malic acid by the action of lactic bacteria. However, lactic acid can also be produced in smaller quantities by the action of yeast already during fermentation [27]. The lactic acid content of the samples varied between 0.56 and 2.04 g·L^−1^. The concentration of lactic acid produced by yeast is in the range of 0.2–0.4 g·L^−1^. More of this acid is produced by the breakdown of malic acid during malolactic fermentation, when its content in wines ranges from 0 to 3 g·L^−1^ [28]. Along with the decrease in lactic acid content, the overall aromatic characteristics of the wine change, which can affect the resulting overall quality of the wine depending on its style [29]. Higher content of lactic acid and lower content of malic acid in red wine samples indicate the course of malolactic fermentation [30].

The density of the wine is an important parameter that is necessary to determine the extract. Wine is a mixture containing mainly dissolved solids (sugars, acids, phenols and mineral salts), which increase its density above the value of the density of pure water. However, it also contains alcohol, whose density is lower than that of water. This means that the density of dry wines is around 0.9 g/cm^3^ and the density of sweet wines with low alcohol content can be around 1.03 g/cm^3^ [31]. According to Michlovský [2], the density of white and red wines is comparable and normally ranges from 0.9912 to 1.0138 g/cm^3^.

A sugar-free extract can be used to characterize the strength of the wine, i.e., the amount of extractive substances. The higher the determined value of the sugar-free extract is, the fuller and stronger the wine is. The values of the sugar-free extract were in the range of 18.77–25.29 g·L^−1^ for the wine samples studied. The minimum amount of sugar-free extract is 16 g·L^−1^ for white wines and 18 g·L^−1^ for red wines (Viticulture and Viticulture Act). All the mentioned analytical values serve to easily and quickly categorize the rated wines according to quality parameters.

### 2.2. FTIR Spectroscopy

**Figure 1:**
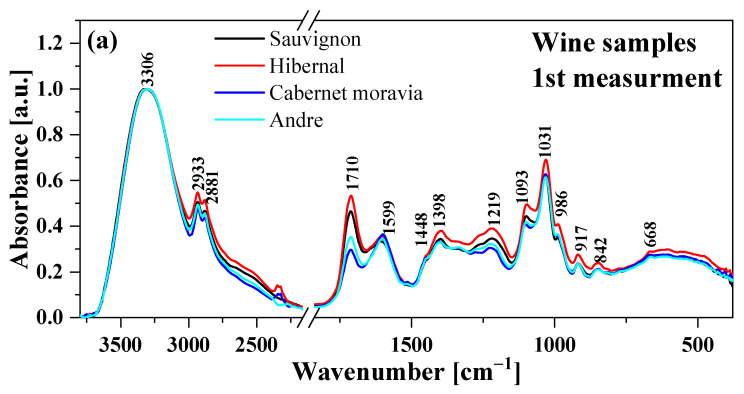

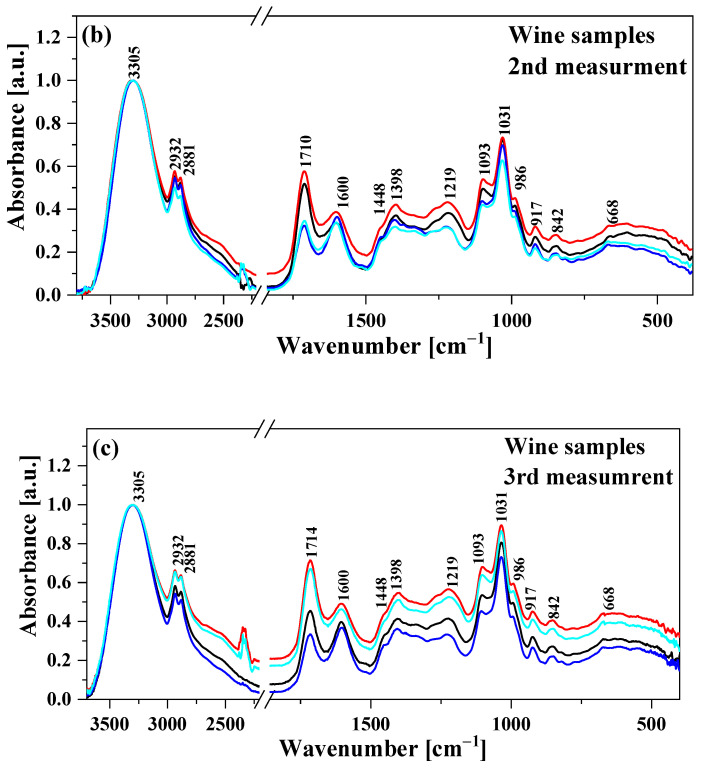
Normalized Fourier transform infrared (FTIR) spectra of wines measured on three different dates. Term I—22.08.22 (**a**), term II—21.10.22 (**b**) and term III—25.11.22 (**c**).

**Figure 2:**
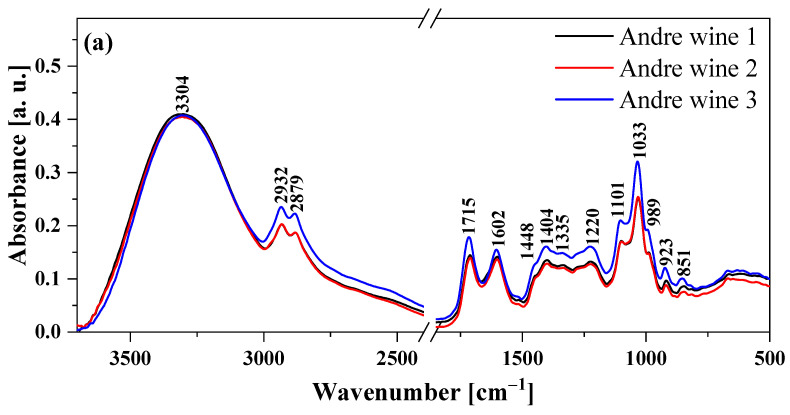

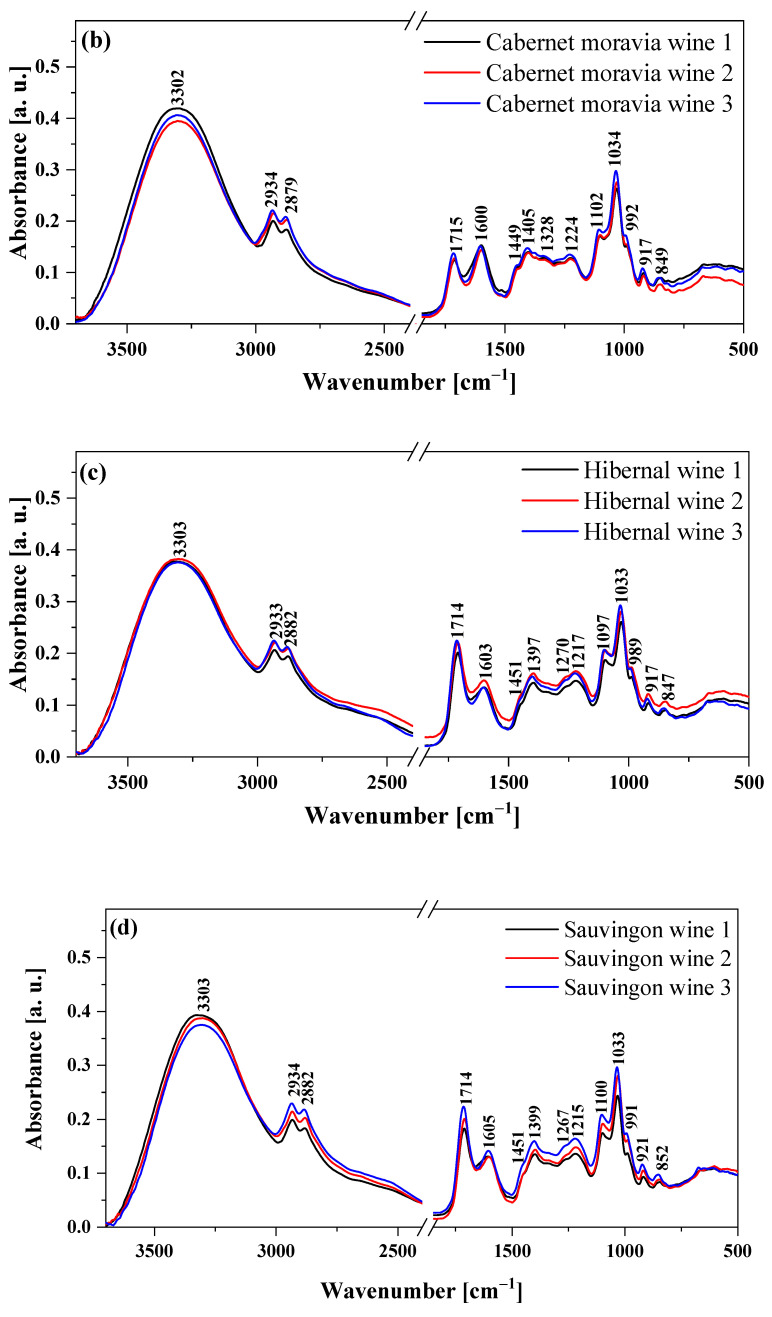
Fourier transform infrared (FTIR) spectra of four types of wines measured on three different dates. André wine (**a**), Cabernet Moravia wine (**b**), Hibernal wine (**c**), Sauvignon wine (**d**).

Figure 1 and Figure 2 show the FTIR spectra of two red wine samples (Andre and Cabernet Moravia wine) and two white wine samples (Hibernal and Sauvignon wine), respectively. The spectra in Figure 1 were normalized to a band present at 3303 cm^−1^. Table 2 presents all the characteristic maxima and assigns the vibrations of the corresponding functional groups. In addition, the selected wine samples were measured at three different time intervals to observe the so-called aging effect (22 August 22, 21 October 22 and 25 November 22), which has a significant impact on wine storage and quality. The results are presented in Figure 2. In the case of all the samples, similar spectral properties were observed. However, when comparing the intensities of some peaks, clear differences between white and red wines were visible.

The FTIR spectra of the wines examined showed absorption bands at various frequencies that were related to the presence of various functional groups that constitute the chemical compounds present in food samples, such as alcohols, phenols, aldehydes, higher alcohols, polyols, sugars and amino acids [32]. All the selected samples had a very broad band in the range of 3800–3000 cm^−1^, resulting mainly from the stretching vibrations of hydroxyl groups, ν(-OH), present in water, alcohol or phenol molecules. The characteristic water absorption bands were present at approximately 990 and 1460 cm^−1^ (stretching and deformation related to the third overtone of these bands) and characteristic deformation bands with maxima at approximately 1600 cm^−1^ [33].

Absorption bands related to the presence of monohydroxy and polyhydroxy alcohols were observed at 2932 and 2880 cm^−1^ (Figure 1, Table 1). These bands correspond to the symmetric and asymmetric stretching vibrations of CH_2_ and CH_3_ groups. The accumulation of these bands, with a wide and noticeably flattened maximum in the range of 2400–2700 cm^−1^, may correspond to a combination of stretching vibrations of C-H and overtones of these vibrations from ethanol molecules and partially from sugar. Additionally, bands in the range of 3000–2800 cm^−1^ are most likely due to the stretching vibrations of C-H bonds present in hydrocarbons and O-H bonds of carboxylic acids, and asymmetric stretching vibrations of C-H bonds of methyl (-CH_3_) groups: polyols (glycerol), free phenolic acids and catechins. In addition, vibrations originating from primary alcohols and glycerol with maxima in the range of 1087 and 1050 cm^−1^, respectively, are related to strong stretching vibrations of C-O [34].

The wavenumbers between 1800 and 1000 cm^−1^ are characteristic of stretching vibrations of C-OH, CH_3_ and CH_2_ deformations and stretching vibrations of C=C and C≡N (Table 1). This region originates from compounds such as phenols, alcohols, aldehydes, higher alcohols, polyols, acids, sugars, volatile acids and amino acids. The range of 1850–1590 cm^−1^ is associated with a combination of stretching vibrations of -OH, -CH_3_ (first harmonics) and -CH_2_, -CH (first harmonics) from ethyl alcohol. Differences in intensity in this region are observed in the range of 1540–1780 cm^−1^, which is related to the presence of amino acids, organic acids and peptides [8].

Bands with maxima at around 1600 and 1700 cm^−1^ (amide I and amide II regions) can be used as a qualitative indicator among various types of wine and their sweetness (sweet, semi-sweet, dry, semi-dry). The intensity ratio of the 1600/1700 cm^−1^ band is different in red and white wines. For André and Cabernet Moravia (red wines), it is 1.01 and 1.21, and for Hibernal and Sauvignon (white wines), it is 0.66 and 0.72, respectively [9].

The region of 1580–950 cm^−1^ (Figure 1) is particularly interesting, as it is characteristic of many compounds present in wines. Therefore, the significant differences observed between the FTIR spectra in this region might be of particular interest in terms of the quality of the wines tested. The region between 1460 and 1280 cm^−1^ is highly complex and provides information on the stretching vibrations of the carbonyl group C=O and the stretching vibrations of C=C, CH_2_ and C-H from molecules of aldehydes, carboxylic acids, proteins and esters. It is also worth emphasizing the bands with maxima around 1220, 1110–1100 and 1070–990 cm^−1^, which correspond to stretching vibrations of C-O and O-H (second harmonics) from sugars and organic acids [8,9].

FTIR spectroscopic analysis showed differences in the regions characteristic of groups related to the presence of alcohols, esters and acids. These differences were most likely related to the grape varieties used. To confirm the origins of the differences between samples and also the aging effects, multivariate analysis techniques such as PCA, HCA and PCA-LDA were performed. Nevertheless, it should be noted that the results obtained clearly indicated the high quality of the selected wine samples for analysis.

#### 2.2.1. Hierarchical Clustering Analysis for FTIR Spectra

**Figure 3:**
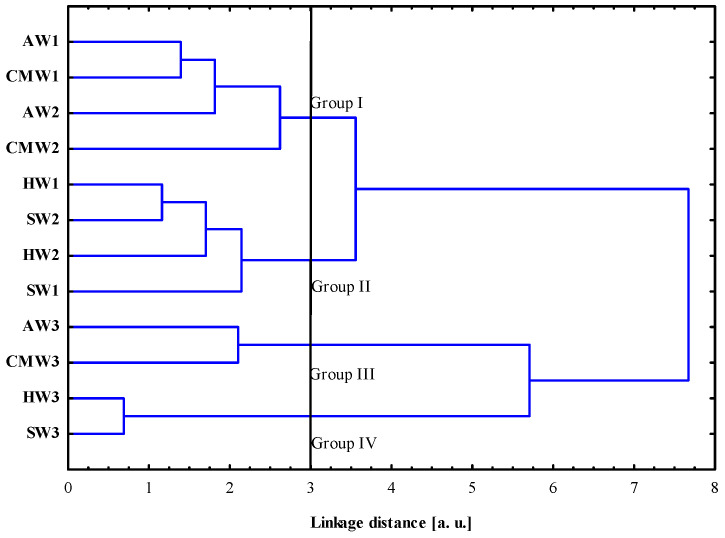
Dendrogram of hierarchical cluster analysis (HCA) of wines based on the Fourier transform infrared (FTIR) spectral data for wavenumber range of 1800–500 cm^−1^. Average linkage and Euclidean distance were used.

HCA was performed on the FTIR spectra to identify similarities or dissimilarities between the considered wine samples. Figure 3 shows the dendrogram obtained from the four wine samples measured at three different times (1—August, 2—October and 3—November). Considering the cut-off of 3.0 dissimilarity units, four clusters were distinguished. The first cluster (Group I) was formed by two types of wine samples (André and Cabernet Moravia measured during 1 and 2 dates), while the second cluster also contained two types of wine samples (Hibernal and Sauvignon measured during 1 and 2 dates). The third and fourth clusters were similar to the first and second clusters but contained samples measured in November. This result suggests that Andre and Cabernet wines as well as Hibernal and Sauvignon wines have physicochemical properties more similar to each other. The hierarchical cluster analysis showed that the placement of the wine samples on the dendrogram depended on the grape variety and sample measurement date during storage.

#### 2.2.2. Principal Component Analysis (PCA) and Linear Discriminant Analysis (LDA) for FTIR Spectra

In order to evaluate the main differences in the FTIR spectra of the different wines, Andre wine (AW), Cabernet Moravia wine (CMW), Hibernal wine (HW) and Sauvignon wine (SW), PCA was applied to region 1800–500 cm^−1^. Then, LDA analysis based on PCA analysis was performed. The contribution of the total variance and the eigenvalues for the selected regions are presented in Table 3. The first three principal components explained almost 94%.

**Figure 4:**
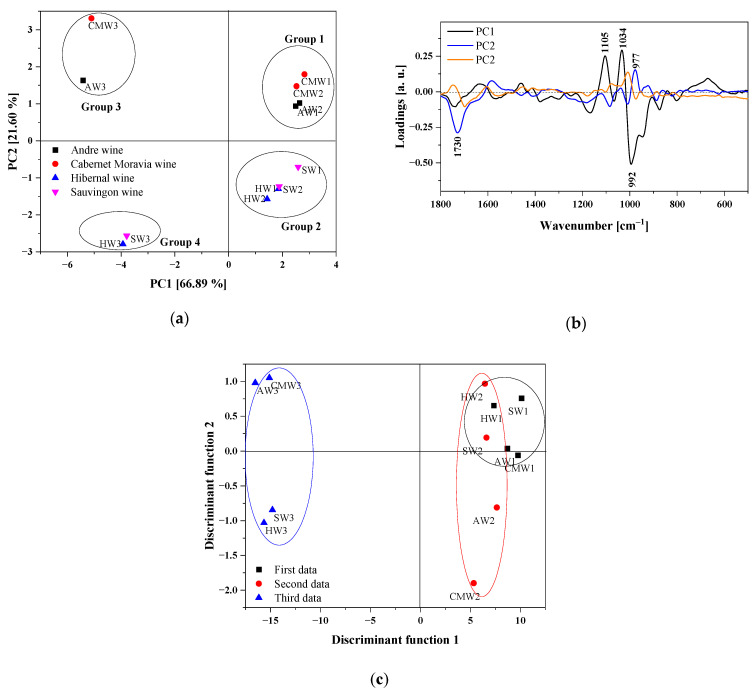
Score plot (PC1 versus PC2) calculated for the data acquired from the FTIR spectra in the range of 1800–500 cm^−^^1^ (**a**). The loading plot of PC1, PC2 and PC3 for the region 1800–500 cm^−^^1^ (**b**). Linear discriminant analysis (LDA) score plot using three PCs as variables (**c**).

As seen in the score plot (Figure 4a), four clusters can be distinguished. The first two consist of samples measured in August and October, while the remaining two are related to samples from November.

The PCA clearly indicates similarities between the Andre, Cabernet Moravia and Hibernal and Sauvignon wines. Secondly, clear differences are present in the spectra of wines recorded at different time intervals. The variables with the greatest influence on the scores were determined based on the loading plot. Moreover, the ordering of variables according to their contributions to the formation of principal components was feasible.

In the PCA loading plot depicted in Figure 4b, PC1 gives evidence for a positive correlation between the stretching vibrations of C-O and O-H groups present in both sugars and organic acids observed at 1105 and 1034 cm^−1^, respectively. These unique features, characteristic of phenols, alcohols, sugars and acids, offer the possibility of the use of the fingerprint regions of the spectra for the classification of various wine samples.

In the next step, the first three principal components were applied as variables in the LDA. The relevant LDA score plot given in Figure 4c represents the first two discriminant functions. It also evidences the achievement of a high degree of discrimination among wine samples, leading to the clear categorization of the wine samples into three groups (I–III). The discriminant model applied for all four samples allowed the correct classification of all samples into their respective groups (I–III), with a success rate of 91.7% (Table 4).

#### 2.2.3. Principal Component Analysis (PCA) for Basic Analytical Values of Evaluated Wine Samples

**Figure 5:**
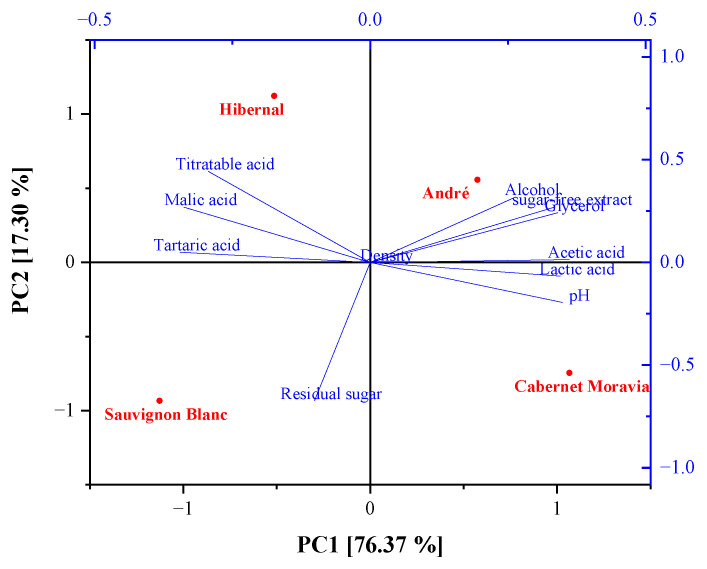
Biplot from principal component analysis (PCA) of the physicochemical composition between white and red wines from the Moravia region.

PCA was performed on the basic physicochemical parameters, corresponding to Table 1, in order to gain insights into the relationship between the analytical data and wine quality. Figure 5 shows a biplot that can be used for the visualization of the results from PCA, as it combines both the principal component scores of the observations on the principal components and the loading vectors to represent the coefficients of the variables in a single 2D plot. The first two principal components explained 93.67% of the variance. Therefore, only the first two components (PC1 and PC2) were used in the further part of the analysis. André and Cabernet Moravia (two blue grape varieties) were separated on the right side of PC1, while Sauvignon Blanc and Hibernal (two white grape varieties) were separated on the left side of PC1. The tested wines showed significant differences in their basic physicochemical compositions (did not form clusters), which was represented by the second principal component, PC2. André wine was characterized by the highest level of alcohol, glycerol content and sugar-free extracts. Moreover, red wines displayed higher lactic and acetic acid content as well as pH compared to white wines. In the case of white wines, they were characterized by higher content of malic, tartaric and titratable acids in relation to red wines. However, there were visible differences in the content of these acids between the two white grape varieties (Sauvignon Blanc and Hibernal). The presented PCA analysis shows that the type of wine (grape variety) and the botanical origin are two factors affecting the quality of wines. Nevertheless, the presented data focus only on the basic physicochemical parameters of the investigated wines. Therefore, in the future, the use of complementary analytical methods should be considered in order to obtain more knowledge of the wines’ quality.

## 3. Materials and Methods

### 3.1. Grape Varieties and Their Origins

For the experimental measurements, a total of four wine samples derived from two white grape varieties (Sauvignon Blanc and Hibernal) and two blue grape varieties (André and Cabernet Moravia) were used. The grapes of the listed varieties were planted and subsequently harvested by hand in a vineyard in the village of Rakvice (48°85′30″ N 16°78′54″ E), Moravia region, Czech Republic. In terms of localization, the region of Moravia (Velké Pavlovice sub-region) within Central Europe is located at the northern edge of the grape-growing area. The average annual temperature is 10 °C, and the long-term average sum of precipitation is 500 mm per year. The average relative humidity is around 80%. The predominant types of soil are chernozem and pelic chernozem; the parent substrate is loess. The soil is silty. They are predominantly represented by moderately heavy, skeletonless soil and very deep soil with a mainly favorable water regime. The depth of the topsoil does not exceed 0.7 m. Soils are located in a very permeable subsoil, mainly skeletonless, medium-dry and dependent on precipitation during the growing season. The mentioned factors create optimal conditions for the growth of mainly aromatic white grape varieties as well as blue Cabernet-type varieties. In addition to soil conditions, the resulting quality of the wines produced is also influenced by the climatic conditions of the given year.

The white varieties were harvested on 30 September 2021, while the blue varieties were harvested on 14 October 2021. The sugar concentration of the harvested grape juice was determined using a normalized saccharimeter and expressed as °NM (kg of total sugar in 100 L of juice). The must sugar content of Sauvignon Blanc grapes was determined at 21.1°NM, that of Hibernal at 20.8°NM, that of André at 22.2°NM and that of Cabernet Moravia at 21.3°NM.

### 3.2. Wine Processing

The white wine production technology included the following steps. Firstly, destemming of the grapes was performed, followed by the maceration of the mash at a temperature of 10 °C for 6 h and pressing with a pneumatic press (at the pressure of 2.2 atm). The pressed must was de-slurried using a vacuum rotary filter. Must fermentation took place in stainless steel tanks with a volume of 600 L, with temperature control at 17 °C for 11 days. At the end of fermentation, the wines were racked, treated with the addition of sulfur dioxide (50 mg/L) and left on fine yeast lees for 2 months. After the second racking, bentonite and free sulfur were added to the level of 40 mg/L. The wines were aged in wooden barrels for 5 months. This was followed by sterile filtration, the addition of free sulfur to the level of 60 mg/L and the filling of wine samples into glass bottles (Bordeaux type) closed with cork caps.

The production technology of red wines included the destemming of grapes and fermentation associated with the maceration of dyes in a vinifier for a total duration of 8 days at a temperature of 22 °C. This was followed by pressing the mash using a pneumatic press (at the pressure of 2.8 atm). The young wines were stored in stainless steel tanks with a volume of 600 L, where malolactic fermentation took place at a temperature of 18 °C. After the wines were racked from the lees and treated with the addition of sulfur dioxide (50 mg/L), they were aged in wooden barrels for 8 months. The production process was completed by adding free sulfur to the level of 60 mg/L, sterile filtration and filling the samples into glass bottles (burgundy type) closed with cork caps.

### 3.3. Determination of Basic Analytical Values in Wine

Total acidity, volatile acidity and pH were determined in the evaluated wine samples according to the standardized methodological procedure of the OIV [35]. A TITROLINE EASY instrument (SI Analytics GmbH, Mainz, Germany) was used to measure these basic analytical values. Residual sugar, acetic acid and sugar free extract concentrations were estimated using a Fourier transform infrared (FTIR) spectrometer (ALPHA) with attenuated total reflection (Bruker Optik GmbH, Ettlingen, Germany). Depending on the calibration used, the measured values were evaluated automatically using the Opus Wine Wizard software. For the determination of glycerol, the colorimetric method of Rebelein was used [36].

### 3.4. ATR-FTIR Measurement

The infrared spectra were recorded on four different wine samples at three different times, namely on 22 August 22, 21 October 22 and 25 November 2022. All spectra were recorded at room temperature (T = 23 °C) using a Shimadzu IRSpirit spectrometer (SHIMADZU, Kyoto, Japan), with a resolution of 4 cm^−1^ and within the range of 4000–300 cm^−1^. The background correction (40 scans per sample) using a dedicated spectrometer extension—the QATR-S Single Reflection ATR Diamond Prism Adapter—was carried out prior to each measurement. All solvents were purchased from Sigma-Aldrich.

### 3.5. Chemometrics Analysis

Multivariate analyses, including hierarchical cluster analysis (HCA), principal component analysis (PCA) and linear discriminant analysis (LDA), were performed for the pre-processed FTIR spectra of wines. Moreover, PCA was carried out also for the physicochemical composition. The Grams/AI 8.0 software (Thermo Scientific, Waltham, MA, USA) was applied for multi-point baseline correction, Savitzky–Golay smoothing, as well as Y offset correlation, and points were set to zero prior to the analysis. Data pre-processing such as mean centering was performed using OriginPro (OriginLab Corporation, Northampton, MA, USA). Chemometrics analysis was conducted in the broad range of spectra 1800–500 cm^−1^. The Statistica 13 software (TIBCO Software Inc., Palo Alto, CA, USA) and OriginPro (OriginLab Corporation, Northampton, MA, USA) were applied for chemometrics analysis.

#### 3.5.1. Hierarchical Clustering Analysis (HCA)

Hierarchical clustering analysis (HCA) is one of the classificatory techniques used for the identification of groups in a dataset [37]. HCA is one of the exploratory methods and consists of dividing the data set into groups in such a way that elements in the same group are similar to each other and, at the same time, as different as possible from elements in other groups. The result is a tree graph called a dendrogram [38]. In HCA, the Euclidean distance between the pairs of samples was used as a distance measurement, and the average linkage criteria were used as an agglomeration method.

#### 3.5.2. Principal Component Analysis (PCA)

Principal component analysis (PCA) is a widely used statistical unsupervised technique to reduce the dimensionality of high-dimensional datasets such as FTIR spectra, while preserving the most important information. It consists of determining a new set of uncorrelated variables, called principal components (PC) [39]. A thorough analysis of the principal components makes it possible to identify those initial variables that have a large impact on the appearance of individual principal components, i.e., those that form a homogeneous group. The principal component (in which the variance is maximized) is then representative of this group. Further components are mutually uncorrelated and are defined so as to maximize the variability that is not explained by the previous component [40].

#### 3.5.3. Linear Discriminant Analysis (LDA)

Linear discriminant analysis (LDA) is a commonly used supervised technique for data classification, dimensionality reduction and data visualization. LDA is used to identify the directions that capture the most separation between the classes by determining a discriminant function [41,42]. Moreover, the LDA method cannot be conducted when the number of spectral variables is larger than the number of samples, so that it can be applied to the extracted PCs from principal component analysis. In this research, LDA was conducted in the 1800–500 cm^−1^ spectral region based on the three first PCs from PCA.

### 3.6. Methods of Statistical Analysis

Results were reported as averages and standard deviations. Analysis of variance (ANOVA) and Tukey’s honestly significant difference (HSD) tests were conducted to determine the differences among averages using the software package Statistica 12.0 (StatSoft Inc., Tulsa, OK, USA). ANOVA was conducted, and the results were compared using Tukey’s multiple range assay at a significance level of α = 0.05.

## 4. Conclusions

In this study, we attempted to establish a classification model of four different wines originating from two white grape varieties (Sauvignon Blanc and Hibernal) and two blue grape varieties (André and Cabernet Moravia) based on their unique fingerprint regions in spectroscopy (FTIR) associated with multivariate analysis (PCA, HCA, PCA-LDA). FTIR analysis showed significant differences between the types of wine examined, particularly within the range of 1540–1780 cm^−1^. Analysis of the PCA loading plots confirmed that the greatest influence on the classification of different samples of wine was attributed to the 1730 cm^−1^, 1105 cm^−1^, 1034 cm^−1^ and 992 cm^−1^ bands, specific to stretching vibrations of C-O, C=O and O-H groups in the sugars and organic acids.

In the context of viticulture, FTIR spectroscopy can be considered a suitable non-destructive technique that provides structural information on the molecular properties of a large range of compounds contained therein. The main advantages of FTIR spectroscopy are its speed, the high degree of automation and economy, which together make it a good prerequisite for routine qualitative analyses and process control in winemaking. The production of wine requires constant monitoring of the product and requires control of the process from the beginning of the ripening of the grapes to the bottling of the finished product. Direct spectroscopic measurement is very suitable in this context, as it allows the rapid and simultaneous analysis of several compounds with minimal sample preparation and reagent consumption. The main prerequisite for the wider application of this method in winemaking practice will be the expansion of commercially available FTIR instrumentation with versatile and innovative software, designed specifically for the analysis of grapes and wine.

Moreover, FTIR spectroscopy can be used as a tool to study the aging processes during food storage, such as those occurring in the wine samples tested. It is extremely important to further understand changes in the physicochemical profiles of foods and to identify a panel of aging biomarkers. Nevertheless, FTIR spectroscopy showed no significant changes in the spectra measured at three different time intervals, which confirmed that the wine products presented fairly high stability.

## Figures and Tables

**Table 1:** The overview of basic analytical values for evaluated wine samples.

		Wine by Variety			
	Unit	Sauvignon Blanc	Hibernal	Cabernet Moravia	André
Alcohol	%	12.19 ± 0.04 ^a^	12.58 ± 0.06 ^b^	12.83 ± 0.07 ^c^	13.58 ± 0.05 ^d^
Titratable acid	g·L^−1^	6.00 ± 0.02 ^a^	6.57 ± 0.29 ^b^	4.74 ± 0.22 ^c^	5.58 ± 0.41 ^d^
Residual sugar	g·L^−1^	1.24 ± 1.12 ^a^	0.00 ± 0.00 ^b^	0.64 ± 0.99 ^c^	0.50 ± 0.44 ^d^
pH	-	3.13 ± 0.04 ^a^	3.15 ± 0.04 ^a^	3.48 ± 0.04 ^c^	3.33 ± 0.06 ^b^
Malic acid	g·L^−1^	3.65 ± 0.17 ^a^	4.01 ± 0.77 ^a^	0.35 ± 0.28 ^b^	1.76 ± 0.41 ^c^
Lactic acid	g·L^−1^	0.56 ± 0.04 ^a^	1.02 ± 0.35 ^ab^	2.04 ± 0.28 ^c^	1.32 ± 0.36b ^c^
Acetic acid	g·L^−1^	0.04 ± 0.09 ^a^	0.17 ± 0.04 ^a^	0.49 ± 0.05 ^b^	0.40 ± 0.07 ^c^
Tartaric acid	g·L^−1^	2.31 ± 0.14 ^a^	2.15 ± 0.12 ^a^	1.83 ± 0.26 ^a^	2.06 ± 0.28 ^a^
Glycerol	g·L^−1^	7.66 ± 0.17 ^b^	8.73 ± 0.73 ^ab^	9.62 ± 0.41 ^a^	9.81 ± 0.31 ^a^
Density	Kg·m^−^³	0.99 ± 0.00 ^a^	0.99 ± 0.00 ^ab^	0.99 ± 0.00 ^b^	0.99 ± 0.00 ^ab^
Sugar-free extract	g·L^−1^	18.77 ± 0.88 ^a^	22.19 ± 0.25 ^ab^	24.59 ± 0.86 ^b^	25.29 ± 2.08 ^ab^

The results are mean values ± SD; analysis of variance (ANOVA) and Tukey’s honestly significant difference (HSD) tests were conducted to determine the differences among averages; values in same column designated with different letters indicate significant differences (*p* ≤ 0.05).

**Table 2:** The positioning of FTIR absorption maxima of the wine samples together with assignment of their corresponding functional groups.

Type and Origin of Vibrations	André Wine	Cabernet Moravia Wine	Hibernal Wine	Sauvignon Wine
ν(O-H) in water and hydroxylated molecules (alcohols and phenols)	3304	3302	3303	3303
ν_w_(-CH) of hydrocarbons	2932	2934	2933	2934
ν_m_(-CH_3_) of hydrocarbons	2879	2879	2882	2882
ν_m_(-C=O)	1715	1715	1714	1714
δ(-OH) and ν(C=C)	1602	1600	1603	1605
ν(C=C) and ν(C-N)	1516	1516	1514	1514
ν(C=C) δ(-CH_3_)	1448	1449	1451	1451
δ_m_(-CH_2_) and δ (-CH)	1404	1405	1397	1399
ν(C=C), δ (-CH_2_)	1335	1330	1332	1332
δ (-CH_2_)	1265	1265	1270	1267
ν_m_(-C-O) and δ_m_(-CH_2_)	1220	1224	1217	1215
ν_st_(-C-O) and ν_w_ (-OH) second overtones	1101	1102	1097	1100
ν_m_(-C-O)	1033 989	1034 992	1033 989	1033 991
δ_w_(-HC=CH-, *trans*-) out-of-plane)	923	917	917	921
δ(-CH_2_-) and -HC=CH-(*cis*-) scissor	851	849	847	852

**Table 3:** Results obtained from principal component analysis: eigenvalues, percentage of variance and cumulative percentage in the data obtained for the wine samples.

Principal Component Number	Eigenvalue	Percentage of Variance (%)	Cumulative (%)
1	11.67722	66.89095	66.8910
2	3.77114	21.60233	88.4933
3	1.07020	6.13044	94.6237
4	0.52446	3.00428	97.6280
5	0.24714	1.41569	99.0437

**Table 4:** LDA prediction matrix.

True Class	Assigned to Class			% Correct Classification
	I	II	III	
I	3	1	0	75%
II	0	4	0	100%
III	0	0	4	100%
Total	3	5	4	91.7%

## Data Availability

The datasets used and/or analyzed during the current study are available from the corresponding author upon reasonable request.

## Sample Availability

Wine samples are available from the authors.
